# Identification of cell context-dependent YAP-associated proteins reveals β_1_ and β_4_ integrin mediate YAP translocation independently of cell spreading

**DOI:** 10.1038/s41598-019-53659-4

**Published:** 2019-11-20

**Authors:** Joanna Y. Lee, Antonia A. Dominguez, Sungmin Nam, Ryan S. Stowers, Lei. S Qi, Ovijit Chaudhuri

**Affiliations:** 1 Department of Mechanical Engineering, Stanford University, Stanford, CA 94305 USA; 2 Department of Bioengineering, Stanford University, Stanford, CA 94305 USA; 3 Department of Chemical and Systems Biology, Stanford University, Stanford, CA 94305 USA; 4 Stanford ChEM-H, Stanford University, Stanford, CA 94305 USA; 5 Present Address: Department of Biochemical and Cellular Pharmacology, Genentech, South San Francisco, CA 94080 USA

**Keywords:** Genetic engineering, Cancer microenvironment, Mechanotransduction, Integrins

## Abstract

Yes-associated protein (YAP) is a transcriptional regulator and mechanotransducer, relaying extracellular matrix (ECM) stiffness into proliferative gene expression in 2D culture. Previous studies show that YAP activation is dependent on F-actin stress fiber mediated nuclear pore opening, however the protein mediators of YAP translocation remain unclear. Here, we show that YAP co-localizes with F-actin during activating conditions, such as sparse plating and culturing on stiff 2D substrates. To identify proteins mediating YAP translocation, we performed co-immunoprecipitation followed by mass spectrometry (co-IP/MS) for proteins that differentially associated with YAP under activating conditions. Interestingly, YAP preferentially associates with β_1_ integrin under activating conditions, and β_4_ integrin under inactivating conditions. In activating conditions, CRISPR/Cas9 knockout (KO) of β_1_ integrin (ΔITGB1) resulted in decreased cell area, which correlated with decreased YAP nuclear localization. ΔITGB1 did not significantly affect the slope of the correlation between YAP nuclear localization with area, but did decrease overall nuclear YAP independently of cell spreading. In contrast, β_4_ integrin KO (ΔITGB4) cells showed no change in cell area and similarly decreased nuclear YAP. These results reveal proteins that differentially associate with YAP during activation, which may aid in regulating YAP nuclear translocation.

## Introduction

There has been increasing evidence that alterations in the physical properties of the extracellular matrix (ECM) drive breast cancer progression^1–3^. Enhanced mammographic density is a key risk factor for breast cancer progression, and alterations in mammographic density arise from changes in the ECM^4–6^. Increased stiffness of the ECM highly correlates with cancer invasion, with approximately a 10-fold increase in stiffness of tumor tissue (~2–5 kPa) compared to normal tissue (~0.1 kPa)^7–9^. *In vitro* studies have shown that culturing mammary epithelial cells (MECs) in collagen-1 (col-1) gels with increased col-1 density, which increases stiffness, induces invasive phenotypes^10,11^. Col-1 binding to β_1_ integrin, β_1_ integrin clustering, and activation of a FAK-Rho-ERK signaling network mediates this invasive phenotype^10,11^. Further, enhanced stiffness alone in a hydrogel containing basement membrane (BM) ligands, absent of col-1, also induces a highly invasive phenotype in MECs^12^. This occurred through a different mechanism, involving BM ligand binding to β_4_ integrin, followed by inhibition of hemidesmosome formation, altered β_4_ integrin signaling, and activation of Rac1 and PI3K by increased stiffness^12^. These data provide compelling evidence that the ECM is a major regulator of BM invasion and ductal carcinoma progression.

YAP (Yes-associated protein), a transcriptional regulator that is deregulated in diverse cancers, has been identified as a key transducer of ECM stiffness in 2D culture but not 3D culture^13–15^. 2D culture studies found that upon culturing MECs on increasingly stiff col-1 coated polyacrylamide (PAM) gel substrates, YAP becomes dephosphorylated, translocates to the nucleus and becomes activated as a transcriptional co-activator^13,16^, regulating expression of genes involved in proliferation and apoptosis^17^. On stiff 2D substrates, perinuclear stress fibers form a cap over the nucleus, flattening the nucleus and stretching nuclear pores, resulting in YAP accumulation in the nucleus^18–20^. Previously, we assayed YAP activation in 2D culture, 3D culture, and *in vivo* and showed that stiffness-induced YAP activation correlates with stress fiber formation and nuclear cross-sectional area^15^. However area alone could not predict YAP nuclear localization^15^. Further, YAP activation was not involved in mediating mechanotransduction in 3D culture, raising the question: what molecular interactions in 2D culture mediate mechanotransduction? Here, we identify proteins that differentially associate with YAP under activating and inactivating conditions, including β_1_ and β_4_ integrin, respectively. CRISPR/Cas9 studies showed that ΔITGB1 (β_1_ integrin) and ΔITGB4 (β_4_ integrin) reduced YAP activation. This reduction in YAP nuclear translocation coincided with both a decrease in cell area and a decrease in the ratio of nuclear/cytoplasmic YAP.

## Results

### Substrate protein composition affects stiffness-induced YAP activation

**Figure 1:**
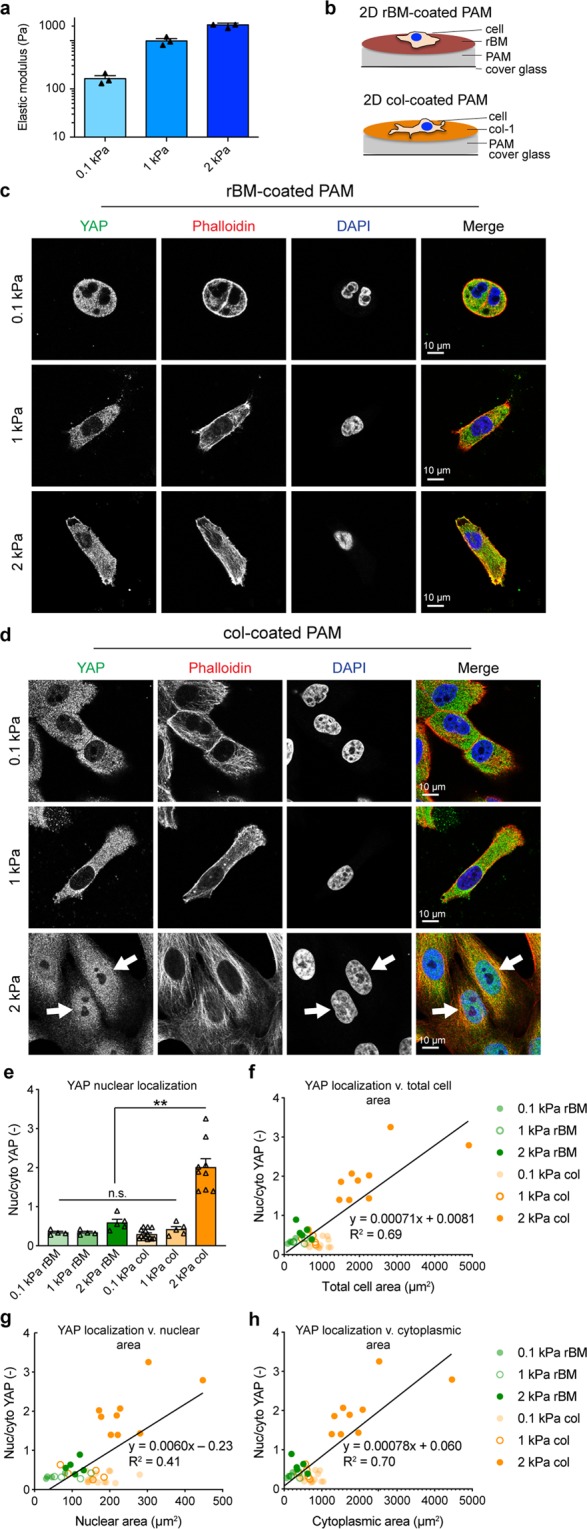
YAP activation depends on 2D substrate stiffness and conjugated surface proteins. (**a)** Unconfined compression of hydrogels at a rate of 1 mm/min was used to obtain the elastic modulus of each hydrogel. Bars represent mean of three gels ± SEM, symbols represent E of each gel. **(b)** Schematic of ligand-coated polyacrylamide (PAM) gels. Confocal micrographs of MCF10A cells plated for 24 h on 2D **(c)** rBM or **(d)** col-coated PAM gels of increasing stiffness. Arrows indicate cells with nuclear YAP localization. YAP (green), F-actin stained by phalloidin (red), DNA stained by DAPI (blue). **(e)** YAP nuclear localization on 2D substrates, **p < 0.0001. Bars represent mean ± SEM, symbols represent each cell, p-values from one-way ANOVA followed by Tukey post-hoc comparison tests. **(f)** Correlation between YAP nuclear localization and total cell area. **(g)** Correlation between YAP nuclear localization and nuclear area. **(h)** Correlation between YAP nuclear localization and cytoplasmic area. N = 4–14 cells from 2 experiments.

To identify the proteins regulating stiffness-induced YAP nuclear translocation, we first established the conditions that induce YAP activation. We generated 0.1, 1, and 2 kPa stiffness 2D PAM gels and conjugated their surfaces to either reconstituted BM (rBM) or col-1 (Fig. 1a,b). Regardless of stiffness, MCF10A cells plated on rBM-coated PAM showed little spreading and YAP localization was cytoplasmic, indicating inactivity (Fig. 1c). However, cells plated on col-1-coated PAM spread with increasing stiffness and 2 kPa stiffness resulted in YAP nuclear localization, consistent with previous studies (Fig. 1d,e and Supplementary Fig. 1)^13^. Increase in YAP nuclear localization corresponded to increased cell area, as measured by increased total cell area (Fig. 1f), nuclear area (Fig. 1g), and cytoplasmic area (Fig. 1h). This is consistent with previous studies showing a relationship between cell area and YAP activation in both 2D and 3D culture^13–15,21–23^. However, these studies cannot distinguish the contributions of spreading v. integrin engagement with substrate surface proteins.

### YAP localizes with F-actin in a context-dependent manner

**Figure 2:**
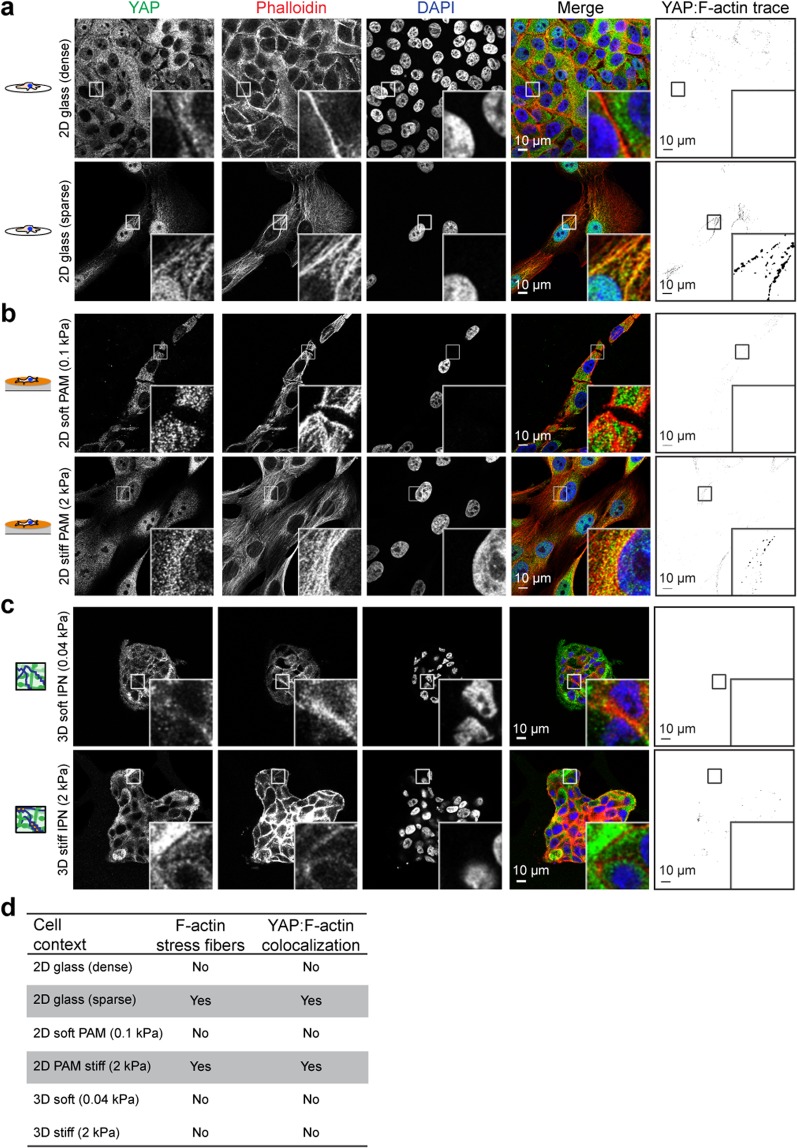
YAP associates with F-actin under activating conditions. MCF10A cells densely or sparsely plated on **(a)** 2D glass coverslips or **(b)** 2D col-1 coated PAM gels, YAP:F-actin analysis in last column indicates overlapping regions of ≥60% YAP pixel intensity and ≥60% F-actin pixel intensity. YAP (green), F-actin stained with phalloidin (red), DNA stained with DAPI (blue). Bar: 10 µm. **(c)** MCF10A cells encapsulated for seven days in soft and stiff IPNs and stained as above. Bar: 10 µm. **(d)** Table of YAP activation in relation to dimensionality and stress fiber formation. Grey boxes indicate conditions that activate YAP.

Limited spreading in cells plated on rBM-coated gels and lower stiffness col-1-coated gels resulted in rounder cells with less surface area, which possessed less prominent F-actin stress fibers, and diminished YAP nuclear localization (Fig. 1c,d), compared to their stiff col-1 coated counterparts (Fig. 1c-e). This is in agreement with previous studies showing the dependence of YAP on F-actin^14,24,25^. As similar cell morphologies are observed in densely and sparsely plated cells, we examined the F-actin stress fiber network with YAP nuclear localization under these conditions (Fig. 2a). As previously described, densely plated MCF10A cells showed cytoplasmic YAP localization while sparsely plated cells possessed nuclear YAP localization^14^. Densely plated cells showed enrichment for cortical F-actin, and a decrease in stress fibers, and sparsely plated cells displayed prominent perinuclear stress fibers (Fig. 2a). Interestingly, YAP colocalized with perinuclear stress fibers in sparsely plated cells, but was excluded from regions of cortical F-actin in densely plated cells. (Fig. 2a). To test if YAP colocalizes with stress fibers in response to stiffness, we imaged MCF10A cells sparsely plated on 2D soft (0.1 kPa) and stiff (2 kPa) PAM gels by confocal microscopy. Our results show YAP-F-actin colocalization in cells plated on stiff PAM gels, but not soft PAM gels, which also correlated with F-actin architecture (Fig. 2b). However, there was a lesser degree of YAP-F-actin colocalization compared to sparsely plated cells on glass. This may be related to diminished stress fiber formation by cells plated on comparably softer PAM compared to glass, which has an elastic modulus on the order of gigapascals. Accordingly, studies that disrupted the F-actin cytoskeleton by pharmacological inhibition do not show YAP-cytoskeletal staining^24^. Similar to cells in dense 2D culture, cells encapsulated in 3D culture in BM-like matrices show predominantly cortical F-actin without visible stress fibers over elastic moduli ranging from 0.04 kPa to 2 kPa (Fig. 2c)^15^. YAP was similarly excluded from regions of cortical F-actin in 3D culture, the predominant F-actin architecture in YAP-inactive 2D and 3D conditions (Fig. 2c). These data are summarized in Fig. 2d.

### Identification of cell context-dependent YAP-associated proteins

**Figure 3:**
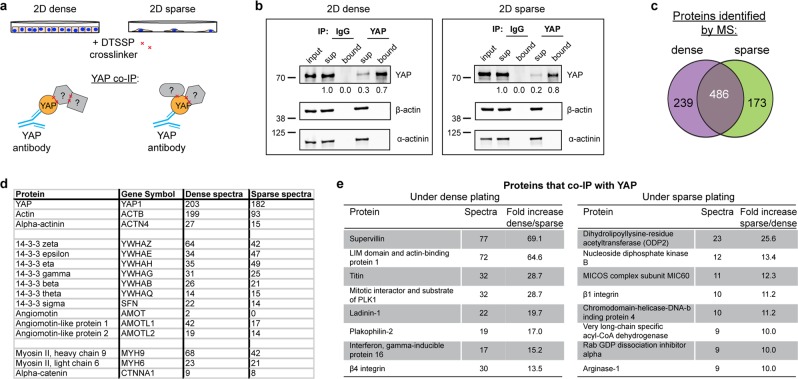
Protein that co-IP with YAP under activating or inactivating conditions. (**a)** Schematic of YAP co-IP from cells crosslinked with 5 mM DTSSP while sparsely or densely plated on 2D tissue culture plastic. **(b)** Western blot analysis of YAP co-IP from cells crosslinked with 5 mM DTSSP while sparsely or densely plated on 2D tissue culture plastic. Bound is 5x load with respect to input. Fluorescence intensity quantification of YAP indicated below each band. Images cropped from the same blot using different fluorescent intensities, stripped, and re-probed. Full-length blot included in Supplementary Fig. 1. **(c)** Venn diagram showing overlap of proteins and number of YAP and actin spectra identified by UPLC-MS/MS to co-IP with YAP under dense or sparse conditions. **(d)** Spectra of YAP, actin, α-actinin, and proteins previously identified as YAP interactors. **(e)** Proteins that differentially co-IP with YAP under sparse or dense conditions.

Given YAP’s reported dependence on stress fibers for activation, and diminished stress fibers in cells cultured in inactivating conditions, we hypothesized that YAP’s nuclear translocation may be mediated by cell context-dependent changes in YAP binding partners, including actin. To identify proteins potentially involved in cell context induced YAP nuclear translocation, we performed a YAP co-IP/MS experiment using sparsely or densely plated MCF10A cells (Fig. 3a). As context-dependent protein binding partners may be lost during the changing cell contexts of trypsinization and lysis, we added a reversible protein crosslinker (DTSSP, 8-atom spacer arm) immediately before cell harvest to ensure protein associations during sparse or dense plating would be preserved. IP of YAP was confirmed by Western blot (WB), using IgG as a control (Fig. 3b and Supplementary Fig. 1a,b) and the bound, YAP IP, fraction subjected to MS. 239 and 173 proteins uniquely associate with YAP under dense and sparse culture conditions, respectively (Fig. 3c). Interestingly comparable actin (ACTB) spectra were identified in both samples (Fig. 3d), at levels greater than that of a common contaminant^26^. This is consistent with the observation of YAP co-localization with stress fibers, and demonstrates that YAP associates with actin in both activating and inactivating conditions. We note that while actin is detected in the bound fraction by MS, the Western blot shows there is not an enrichment of actin in the vicinity of (i.e. associating with) YAP relative to the unbound fraction (Fig. 3b); this may reflect low levels of bound F-actin relative to the abundant pool of unbound G-actin. The MS results also show that YAP differentially associates with β_4_ integrin under dense, inactivating, plating conditions and β_1_ integrin under sparse, activating conditions (Fig. 3e). While integrins are known to function upstream of YAP during mechanotransduction this is the first implication that YAP associates directly with integrins. Several actin-associated proteins also co-IPed with YAP, including supervillin and LIMA1 (Fig. 3e). As expected, canonical YAP binding proteins, including 14-3-3, angiomotin, myosin II, and α-catenin, were found to bind to YAP independently of YAP activation state (Fig. 3d). These data show that changes in cell context alter the complement of YAP-associated proteins, which may facilitate its nuclear translocation.

### CRISPR/Cas9 KO of integrins lowers nuclear/cytoplasmic YAP ratio

**Figure 4:**
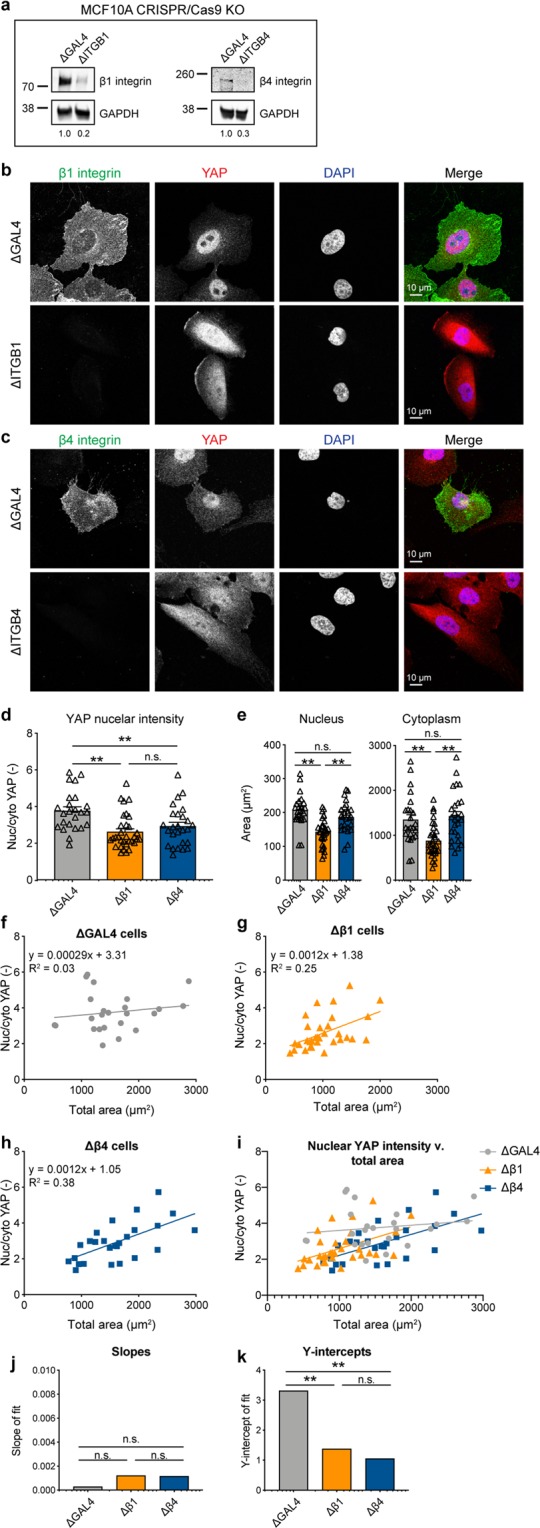
β_1_ and β_4_ integrin KO decrease YAP nuclear localization. (**a)** Western blot analysis of MCF10A ΔITGB1 and ΔITGB4 cells compared to control ΔGAL4 cells. GAPDH was used as a loading control. Quantification of bands (β_1_ or β_4_ integrin/GAPDH) below each lane. Images cropped from the same blot using different fluorescent intensities. Full-length blot included in Supplementary Fig. 2. **(b)** ΔITGB1 cells stained for β_1_ integrin (green) and YAP (red), DNA stained with DAPI (blue). **(c)** ΔITGB4 cells stained for β_4_ integrin (green) and YAP (red), DNA stained with DAPI (blue). **(d)** YAP nuclear localization in KO cells, **p < 0.01. **(e)** Nuclear and cytoplasmic areas of KO cells, **p < 0.002. Bars represent mean ± SEM, symbols represent each cell, p-values from one-way ANOVA followed by Tukey post-hoc comparison tests. **(f)** ΔGAL4 YAP nuclear localization with area. **(g)** ΔITGB1 YAP nuclear localization with area. **(h)** ΔITGB4 YAP nuclear localization with area. N = 24–32 cells from 3 independent experiments. **(i)** Comparison of fits for YAP nuclear localization with area in KO cells. Comparison of **(j)** Slopes and **(k)** y-intercepts from best fit lines of YAP nuclear localization with area in KO cells from (**f**–**h**). **p < 0.01. p-values from linear regression comparing 3 KO best fit lines.

As our co-IP/MS results implicate β_1_ and β_4_ integrins as proteins that differentially associate with YAP, and integrins regulate sensing of ECM stiffness, we generated CRISPR/Cas9 KO MCF10A cells to examine if these integrins mediate YAP nuclear translocation. KO of β_1_ or β_4_ integrin was confirmed by WB, showing 80% and 70% depletion of the proteins compared to control ΔGAL4 cells, respectively (Fig. 4a, and Supplementary Fig. 2a,b). KO was further confirmed by IF staining for β_1_ and β_4_ integrin (Fig. 4b,c). ΔITGB1 cells showed decreased YAP nuclear localization compared to control ΔGAL4 cells (Fig. 4b,d), in agreement with previous studies using β_1_ KO cells^27^. ΔITGB4 cells also showed a slight, but significant, change in YAP nuclear accumulation (Fig. 4c,d). Further, ΔITGB1 cells showed significant decreases in nuclear and cytoplasmic area, while ΔITGB4 cells did not (Fig. 4e), indicating that β_4_ integrin affects YAP nuclear localization independently of cell spreading.

We considered that ΔITGB1 cells may also affect YAP localization beyond its role in cell spreading. To test if ΔITGB1 modulates YAP activation beyond its inhibition of cell spreading, we analyzed YAP translocation as a function of cell area in ΔGAL4, ΔITGB1, and ΔITGB4 cells (Fig. 4f–i). Linear regressions for neither ΔITGB1 nor ΔITGB4 showed a significant change in slope (p = 0.11; Fig. 4i,j). However, linear regressions for both ΔITGB1 and ΔITGB4 showed a significant change in y-intercept (p = 0.0023; Fig. 4i,k). While the slopes are not significantly different, the y-intercepts are, therefore ΔITGB1 and ΔITGB4 cells have significantly less YAP nuclear intensity than ΔGAL cells independent of cell area. This suggests that β_1_ and β_4_ integrin regulate YAP nuclear localization beyond their effects on cell morphology. Together, these data provide compelling evidence that β_1_ and β_4_ integrin mediate context-dependent YAP translocation independently of cell spreading.

## Discussion

YAP is widely accepted as the mechanotransducer of ECM stiffness in 2D culture. While the requirement of stiffness-induced nuclear pore opening for YAP nuclear accumulation has been uncovered, the proteins mediating YAP nuclear translocation during stiffness sensing are not well understood. Using co-IP/MS we identified a biochemical association of YAP with actin in both sparsely and densely plated cells. This is consistent with IF results showing YAP co-localization with F-actin stress fibers in sparsely plated cells or cells on stiff PAM gels. The exclusion of YAP from the cortical actin network in densely plated cells in 2D culture or 3D cultured cells suggests that YAP may interact primarily with G-actin in the absence of stress fibers. The co-IP/MS results also reveal proteins that differentially associated with YAP during its active and inactive states, including supervillin, LIMA1, and ODP2, which may provide further insight into key players in the YAP-dependent mechanotransduction machinery.

A key finding of this work is that YAP can associate with β_1_ and β_4_ integrins in a context dependent manner, and mediate YAP nuclear translocation beyond their effects on cell area. The roles of β_1_ and β_4_ integrins^12,28–30^ and YAP^14,15,31–34^ in breast cancer invasion have been independently studied extensively, however their interaction has only begun to be investigated^27,35–37^. As integrins connect the intracellular cytoskeleton to the ECM, they have been heavily investigated for their role in communicating ECM stiffness sensing^27,38–40^. Our co-IP/MS findings that YAP associates with β_1_ integrin and β_4_ integrin in a cell context-dependent manner are novel. This is in agreement with previous findings that β1 integrin mediates YAP activity^27,36,37,41^. Indeed, it has been shown that cell spreading mediates YAP activity, and it is known that β_1_ integrin mediates cell spreading, providing an explanation for the role of β_1_ integrin in YAP activity^13,42,43^. However, our finding that the correlation between cell spreading and nuclear/cytoplasmic ratio of YAP is altered in ΔITGB1 cells, specifically the y-intercept, indicates that β_1_ integrin may mediate its translocation beyond its role in simply regulating cell spreading. Broadly, the identification of YAP-protein interactions in activating and inactivating conditions provides a rich set of data to guide new studies of molecular mechanisms underlying YAP-mediated mechanotransduction in 2D culture.

## Methods

### Cell culture and cell line

MCF10A cells obtained from the ATCC (cat. #CRL-10317; ATCC) were cultured in DMEM/F12 50/50 medium (cat. #11330057; Thermo Fisher Scientific) supplemented with 5% horse serum (cat. #16050122; Thermo Fisher Scientific), 20 ng/ml EGF (cat. #AF-100-15; Peprotech, Inc.), 0.5 µg/ml hydrocortisone (cat. #H0888-1G; Sigma), 100 ng/ml cholera toxin (at. #C8052-1MG; Sigma), 10 µg/ml insulin (cat. #91077C-250MG; Sigma), and 100 U/ml Pen/Strep (cat. #15140; Thermo Fisher Scientific) as described in^44^. For sparse/dense cell plating, 1–20 mm polylysine coated coverslips were placed in each well of a 12-well TC dish, UV sterilized, seeded with 1 × 10^5^ (sparse) or 1 × 10^6^ (dense) MCF10A cells, and incubated for 24 h to allow attachment.

For inducible MCF10A::Cas9 cell line, lentivirus was produced harboring Edit-R Inducible Lentiviral hEF1α-Blast-Cas9 Nuclease Plasmid DNA (cat. #CAS11229; Dharmacon) (see Cloning and lentiviral generation below). Following infection, Cas9 cells were maintained in MCF10A growth medium as above, supplemented with 5 µg/ml blasticidin (cat. #R21001; Thermo Fisher Scientific). Following a second round of infection with indicated sgRNAs (see Cloning and lentiviral generation below), MCF10A::Cas9/sgRNA cell lines were maintained in medium supplemented with 5 µg/ml blasticidin and 1 µg/ml puromycin (cat. #A1113803; Thermo Fisher Scientific). CRISPR/Cas9 editing was induced by adding 2 µg/ml dox (cat. #AAJ6042206; Alfa Aesar). KO cells were then subjected to expression-based selection using antibodies against β1 and β4 integrin, where the negative population was FACS sorted and collected. Knockout was then verified by WB and IF.

### Cloning and lentiviral generation

All plasmids in this study have been banked in Addgene using the following ID numbers:

pLenti-GAL4 (121514), pSLQ1371-sgITGB1-1 (121533), pSLQ1371-sgITGB1-3 (121534), pSLQ1371-sgITGB4-1 (121535), pSLQ1371-sgITGB4-3 (121536).

sgRNAs were expressed using a lentiviral mouse U6 (mU6) promoter-driven expression vector that coexpessed Puro-T2A-mCherry from a CMV promoter. sgRNA sequences were generated by PCR and introduced by InFusion cloning into the sgRNA expression vector digested with BstXI and XhoI. For each target 2 sgRNA sequences were used and pooled during lentiviral production. sgITGB1-1 sequence: GAAGCAGGGCCAAATTGTGGG; sgITGB1-3 sequence: GTTCAGTGAATGGGAACAACG; sgITGB4-1: GAGGCGCAGTCCTTATCCACA; sgITGB4-3: GAGGAGCGTAGGTCCTCGCAG.

For lentiviral generation, HEK293T cells were seeded at 1 × 10^7^ cells/10 cm dish. The next day 70–90% confluent cells were transfected. For each dish, 9 ug of lentiviral transfer vector, 8 μg of packaging vector pCMV-dR8.91 and 1 ug of packaging vector pMD2-G were transfected using Lipofectamine 3000 Transfection Reagent (cat. #L3000008; ThermoFisherScientific) Opti-MEM Reduced Serum Medium (cat. #31985062; Gibco) according to the manufacturer’s instructions. Medium was replaced with complete medium 4 h following transfection. 48 h following transfection, lentivirus-containing supernatant was harvested and filtered through a 0.22 μm Steriflip (Millipore). Filtered supernatant was concentrated using Lentivirus Precipitation Solution (cat. #VC100; AlStem) according to the manufacturer’s instructions. Following concentration, lentiviral pellets were resuspended in 1/100 of original volume using cold DMEM/F12 and stored at −80 °C. For MCF10A transduction, concentrated lentivirus was added to complete medium containing 8 µg/ml polybrene (cat. #SC134220; Santa Cruz Biotech) at a volume of 1:100.

### Hydrogel formation

Matrigel (cat. #354230; Corning) was purchased for use as rBM matrix and used at a final concentration of 4.4 mg/ml for all experiments. Collagen-1, derived from rat tail, (cat. #354236; Corning) was lyophilized and reconstituted in 20 mM acetic acid. Immediately before cell encapsulation, reconstituted col was supplemented with 10x PBS, neutralized with 0.1 M NaOH, and pH adjusted with 0.1 N HCl. rBM and col were mixed with cells and DMEM/F12 to the reach the indicated final concentrations. MCF10A cells were trypsinized, strained through a 40 µm cell strainer to enrich for single cells, counted on a Vi-CELL (Beckman Coulter Life Sciences), and seeded at a final concentration of 1 × 10^5^ cells/ml hydrogel. Hydrogel-cell mixtures were quickly deposited into wells of a 24-well plate pre-coated with 50 µl gelled rBM. Hydrogels containing cells were placed in a 37 °C incubator with CO_2_ to gel for 30 min before a transwell insert (Millipore) was placed on top to prevent floating and 1.5 ml complete medium added.

IPNs were formed as described^12^. Briefly, LF20/40 alginate (FMC Biopolymer) was solubilized, dialyzed, charcoal filtered, sterile filtered, lyophilized, and reconstituted to 2.5% w/v in DMEM/F12. Alginate was mixed with rBM, cells, and DMEM/F12 and loaded into a 1 ml Luer lock syringe (Cole-Parmer), on ice. For crosslinking, a second 1 ml syringe was loaded with 125 mM CaSO_4_ or DMEM/F12, on ice. Syringes were connected with a female-female Luer lock coupler (ValuePlastics), rapidly mixed with 4–6 pumps of the syringes handles back and forth, and quickly deposited into pre-coated wells, as above. IPNs containing cells were allowed to gel before adding transwell filters and medium, as above.

For 2D PAM gels, the surface of coverslips was functionalized accoring to a previous method^45^. Coverslips were cleaned with ethanol, immersed in 0.5% (3-Aminopropyl)trimethoxysilane (in dH_2_O) at room temperature for 30 min and washed with dH_2_O. Coverslips were then immersed in 0.5% glutaraldehyde in dH_2_O at room temperature for 30 min and dried. A prepolymer solution was prepared containing acrylamide, N,N′-methylene-bis-acrylamide, 1/100 volume of 10% Ammonium Persulfate (APS), and 1/1000 volume of N,N,N′,N′-Tetramethylethylenediamine (TEMED). The final concentration of acrylamide and bis-acrylamide was varied to control substrate stiffness^46^. For 0.1 kPa hydrogels, 3% /0.02% were used. For 1 kPa hydrogels, 3% /0.1% were used. For 2 kPa hydrogels, 4% /0.1% were used. Prepolymer solutions were deposited on a Sigmacote-treated hydrophobic glass plate, and functionalized coverslips placed on top of the prepolymer solution. Polyacrylamide solutions were allowed to polymerize for 30 minutes between the hydrophobic glass plate and the functionalized coverslip. When polymerization was completed, polyacrylamide gels were carefully separated from the glass plate.

To enable cell adhesion to the PAM gel, col-1 and rBM were conjugated to the gel surface using sulfosuccinimidyl 6-(4′-azido-2′-nitrophenylamino)hexanoate (sulfo-SANPAH) as a protein-substrate linker. PAM gels were incubated in 1 mg/ml sulfo-SANPAH in 50 mM HEPES pH 8.5, activated with UV light (wavelength 365 nm, intensity 4 mW/cm^2^) for 20 min, washed in HEPES, and then incubated in 100 ug/ml col-1 and rBM in HEPES overnight at room temperature. The protein-crosslinked PAM gels were washed with PBS before use.

### Mechanical testing

Stiffness measurements of 3D culture rBM, col, and IPN hydrogels were performed using an AR-G2 stress-controlled rheometer with 25-mm top- and bottom-plate stainless steel geometries (TA Instruments). Hydrogel solutions without cells were mixed and immediately deposited onto the bottom plate of the rheometer and the top plate lowered such that the gel formed a uniform disk between the two plates. Exposed hydrogel surfaces were coated with mineral oil (Sigma) and covered with a hydration chamber to prevent sample dehydration. The storage modulus was monitored at 37 °C with 1% strain at a frequency of 1 Hz and measurements taken once the storage modulus reached an equilibrium value. The storage and loss moduli were then used to calculate the Young’s modulus (E). Young’s moduli (i.e. elastic moduli) were calculated using the equation $E=2{G}^{\ast }\times (1+\nu )$, where $\nu$ is Poisson’s ratio, assumed to be 0.5, and ${G}^{\ast }$ is the bulk modulus calculated using the equation ${G}^{\ast }={(G^{\prime2}+G^{{\prime\prime}2})}^{1/2}$ where ${G^{\prime} }^{2}$ is the storage and $G^{{\prime\prime}2}$ is the loss modulus.

To measure substrate stiffness of 2D PAM gels, unconfined compression tests were performed using an Instron MicroTester 5848. PAM gels were compressed at a rate of 1 mm/min. The tangent elastic modulus of the measured stress-strain curves was calculated between 5–15% strain^7,10^. Stiffness of 3D culture alginate hydrogels was measured using unconfined compression tests according to a previously published method^43^. Briefly, alginate disks (15 mm in diameter, 2 mm thick) were submerged in DMEM for 1 day to fully equilibrate. The gel disks were compressed to 15% at a rate of 1 mm/min and the slope of the stress-strain curve from 5% to 10% strain was used to obtain the stiffness of alginate hydrogel.

### Antibodies

Mouse anti–YAP (cat. #sc-101199; Santa Cruz Biotech) was used at 1:200 (IF) and 1:500 (WB). Rabbit anti-p38 (cat. #sc-535; Santa Cruz Biotechnology) was used at 1:2000 for WB. Rabbit anti-α-actinin (cat. #3134; Cell Signaling Technology) was used at 1:500 for WB. Rabbit anti-β-actin (cat. #8457; Cell Signaling Technology) was used at 1:1000 for WB. Phalloidin-Alexa555 (A34055; Thermo Fisher Scientific) was used at 1:100 and DAPI (cat. # D9542; Sigma-Aldrich) was used at 5 µg/ml for IF. Mouse anti-β1 integrin (cat. #ab24693, Abcam) was used at 1:200 for IHC. Mouse anti-β1 integrin (cat. #sc-374429, Santa Cruz Biotechnology) was used at 1:1000 for WB. Rat anti–β4 integrin (cat. # 14-1049-82; Thermo Fisher Scientific) was used at 1:500 (IF) and 1:1000 (WB).

For IF, Alexa 488-, 555- or 647-conjugated secondary antibodies (Thermo Fisher Scientific) were used at 1:500. For WB, IRDye 680 or 800-conjugated secondary antibodies (LI-COR Biotechnology) were used at 1:10,000.

### Western blotting, immunoprecipitation, and mass spectrometry

Uncropped WBs are shown in Supplementary Figs 2 and 3. MCF10A cells encapsulated for 7 days were harvested from IPNs by incubation in cold PBS containing 50 mM EDTA (Sigma) for 5 min while pipetting to break up gels. Cells were centrifuged at 500 x g for 10 min. The supernatant was removed and the cells with remaining matrix material were treated with 0.25% trypsin (Gibco) for 5 min and centrifuged for 5 min at 500 × g. Cell pellets were washed with 20% serum-containing resuspension buffer to neutralize trypsin and washed twice with PBS. For SDS-PAGE of whole cell lysates, MCF10A cells were lysed in Pierce RIPA buffer (cat. #89900; Thermo Fisher Scientific) supplemented with Protease Inhibitor Cocktail Tablets (cat. #11836170001; Roche) and PhosSTOP Phosphatase Inhibitor Cocktail Tablets (cat. #04906845001; Roche) according to the manufacturer’s instructions. Protein concentration was determined using the Pierce BCA Protein Assay Kit (cat. #23227; Thermo Fisher Scientific). Laemmli sample buffer (cat. #1610747; Bio-Rad) was added to lysates and samples boiled for 10 min before loading 25 µg protein in each lane of a 4–15%, 15-well, gradient gel (cat. # 4561086; Bio-Rad). Proteins were transferred to nitrocellulose at 100 V for 105 min, blocked with 5% milk in TBS-T (137 mM NaCl, 2.7 mM KCl, 19 mM Tris base, 0.1% Tween, pH 7.4), incubated in primary antibody overnight, and fluorescent secondary antibody for 1 h. Blots were visualized using IRDye 680- or 800-conjugated secondary antibodies with the Li-COR Odyssey imaging system (Li-COR Biotechnology). Quantitative analysis of western blots was performed using the Li-COR Odyssey software (LI-COR Biotechnology).

For IPs, MCF10A cells were harvested from IPNs as above and lysed in Pierce IP buffer (cat. #87787; Thermo Fisher Scientific) containing inhibitors as above and quantified as above. 1 µg of antibodies were conjugated to 10 µl Dynabeads (cat. # 10001D; Thermo Fisher Scientific), added to cell lysate, and rotated for 30–60 min on ice. Beads were washed 3x with IP buffer containing inhibitors followed by elution of protein complexes off beads with sample buffer.

For mass spectrometry, IP samples were validated by WB as above and then subjected to SDS-PAGE. Gels were fixed for 30 min in Coomassie fixative (50% methanol, 10% glacial acetic acid), gel lanes excised, and submitted for mass spectrometry. UPLC-MS/MS was completed using nanoACQUITY UPLC columns (Waters) in front of Orbitrap Elite mass spectrometers (Thermo Fisher Scientific). For determining proteins with greatest fold increase in dense/sparse and sparse/dense, the ratio of protein spectra was calculated and common contaminants (proteins found in greater than 20% of negative control data sets)^26^ excluded.

### Immunofluorescence

Cells encapsulated in hydrogels for seven days were fixed for 30 min in 4% paraformaldehyde in DMEM/F12. Gels containing cells were washed with PBS and incubated in 30% sucrose in PBS with calcium and magnesium overnight followed by incubation in 50/50 30% sucrose/OCT for 6 h. Gels were embedded in OCT and frozen prior to cutting 40 µm sections using a Microm HM 550 Cryostat. Sections were blocked in PBS-BT+: PBS pH 7.4 (Gibco) supplemented with 1% BSA (Sigma-Aldrich), 0.1% Triton X-100 (Sigma-Aldrich), 0.3 M glycine (Sigma-Aldrich), 10% goat serum (Gibco), and 0.05% sodium azide (Sigma-Aldrich). Sections were incubated in primary antibodies diluted in blocking solution as indicated in “antibodies” section for 1 h, and then Alexa 488-, 555- or 647-conjugated secondary antibodies (Thermo Fisher Scientific) diluted 1:500 in blocking solution for 30 min. Sections of gels containing cells were imaged using a Leica TCS SP8 confocal laser scanning microscope (Leica Microsystems, Inc.) with an HC PL APO 63× (1.40 NA Oil CS2) objective. Images were collected from HyD and PMT detectors using LasX software and processed using Photoshop (Adobe Systems).

For YAP-F-actin co-localization traces, MatLab was used to analyze YAP and phalloidin images and generate a new image that marks every pixel that contains both ≥60% YAP pixel intensity and ≥60% F-actin pixel intensity.

Image analysis of YAP nuclear/cytoplasmic intensity and nuclear morphology were performed as previously described^15^. Cell Profiler was used to quantify YAP nuclear/cytoplasmic intensity with the Cell Profiler “Colocalization” pipeline using phalloidin as the cytoplasmic marker and DAPI as the nuclear marker. ImageJ was used to determine nuclear morphology (e.g. area) metrics for each cell and its corresponding nuclear YAP intensity by first tracing cell nuclei using DAPI images following the macro below (pixel/µm of image was first determined and replaced in “Set Scale” distance). Doublets or cell debris were manually excluded. Nuclear traces were then overlaid on YAP images to measure mean nuclear YAP intensity using the following macro.

Macro to trace cell nuclei:

run(“Set Scale…”, “distance = [3.45] known = 1 pixel = 1 unit = µm”);

run(“Gaussian Blur…”, “sigma = 2”);

run(“Subtract Background…”, “rolling = 50”);

setAutoThreshold();

//run(“Threshold…”);

setAutoThreshold();

setThreshold(55, 255);

run(“Convert to Mask”);

run(“Fill Holes”);

run(“Watershed”);

run(“Find Edges”);

run(“Analyze Particles…”, “size = 100-Infinity pixel circularity = 0.00–1.00 show = Nothing exclude add”);

close();

Macro to measure nuclear YAP intensity:

run(“Set Measurements…”, “area mean center perimeter bounding shape integrated skewness redirect = None decimal = 3”);

run(“Set Scale…”, “distance = [3.45] known = 1 pixel = 1 unit = µm”);

setOption(“Show All”,true);

roiManager(“Measure”);

saveAs(“Measurements”, “/Users/Joanna/Desktop/Results.xls”);

### Statistical analysis

Statistical parameters are reported in Figure Legends. Statistical analyses were conducted with one-way ANOVA followed by Tukey post-hoc comparison tests using 3 independent trials unless otherwise noted. Slopes and intercepts of lines were compared using linear regression. Values with p < 0.05 were considered statistically significant.

## Supplementary information

Supplementary Information

## Data Availability

MS proteomics data are stored in PRIDE with the accession code PXD007287.
