# Supplementary material for: Identification of cell context-dependent YAP-associated proteins reveals β1 and β4 integrin mediate YAP translocation independently of cell spreading
Source: Sci Rep. 2019 Nov 20;9:17188. doi: 10.1038/s41598-019-53659-4 (PMC6868278; doi:10.1038/s41598-019-53659-4)
Supplement: Supplementary file 1 — Supplementary Information [file 41598_2019_53659_MOESM1_ESM.pdf]

Supplementary Information

Identification of cell context-dependent YAP-associated proteins reveals β_1 and β_4 integrin mediate YAP translocation independently of cell spreading

Joanna Y. Lee[1+], Antonia A. Dominguez[2-4], Sungmin Nam[1], Ryan S. Stowers[1], Lei S. Qi[2-4], and Ovijit Chaudhuri[1*]

Affiliations:
[1]Department of Mechanical Engineering, Stanford University, Stanford, CA 94305

[2]Department of Bioengineering, Stanford University, Stanford, CA 94305.

[3]Department of Chemical and Systems Biology, Stanford University, Stanford, CA 94305.

[4]Stanford ChEM-H, Stanford University, Stanford, CA 94305.

[+]Current affiliation: Department of Biochemical and Cellular Pharmacology, Genentech, South San Francisco, CA 94080

*Correspondence to: chaudhuri@stanford.edu

Includes:
Supplementary Figures 1–3

Supplementary Figures

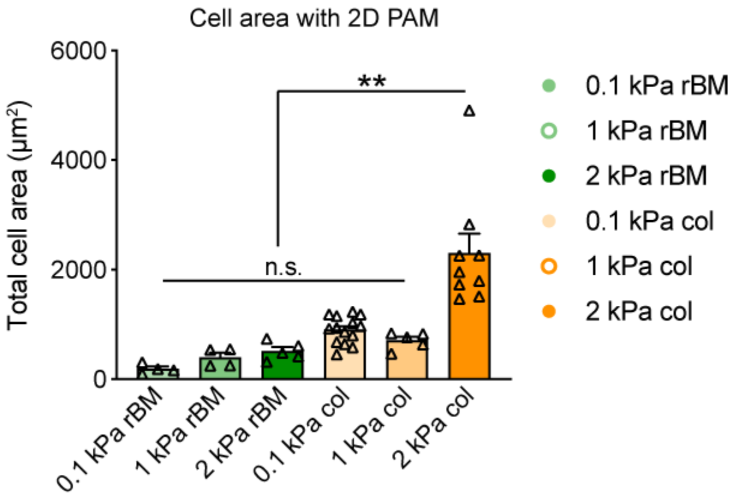

Supplementary Figure 1. Cell area with 2D PAM conditions. Total cell area (i.e. nuclear + cytoplasmic) on 2D substrates, $^{**}p < 0.0001$. Bars represent mean ± SEM, symbols represent each cell, p-values from one-way ANOVA followed by Tukey post-hoc comparison tests.

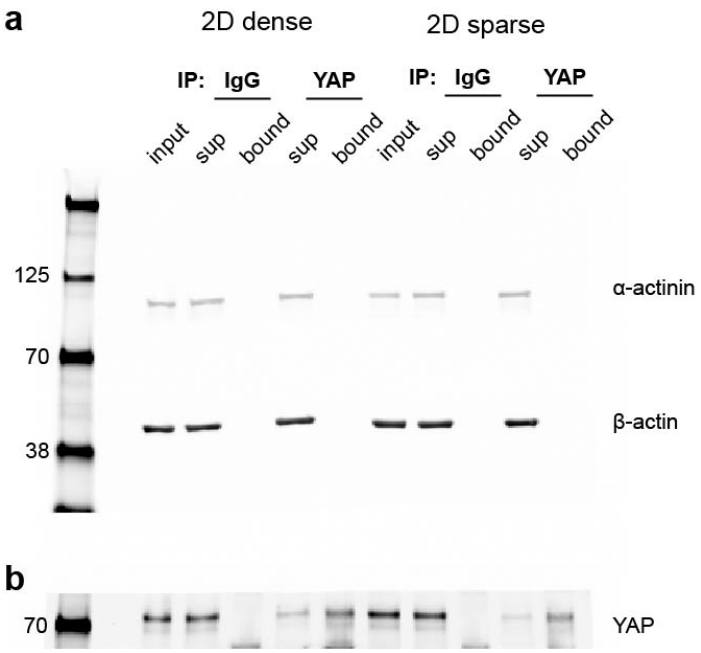

Supplementary Figure 2. Uncropped Western blots from co-IP. (a) α-actinin and β-actin blots. **(b)** Blot from (a) stripped and re-probed for YAP.

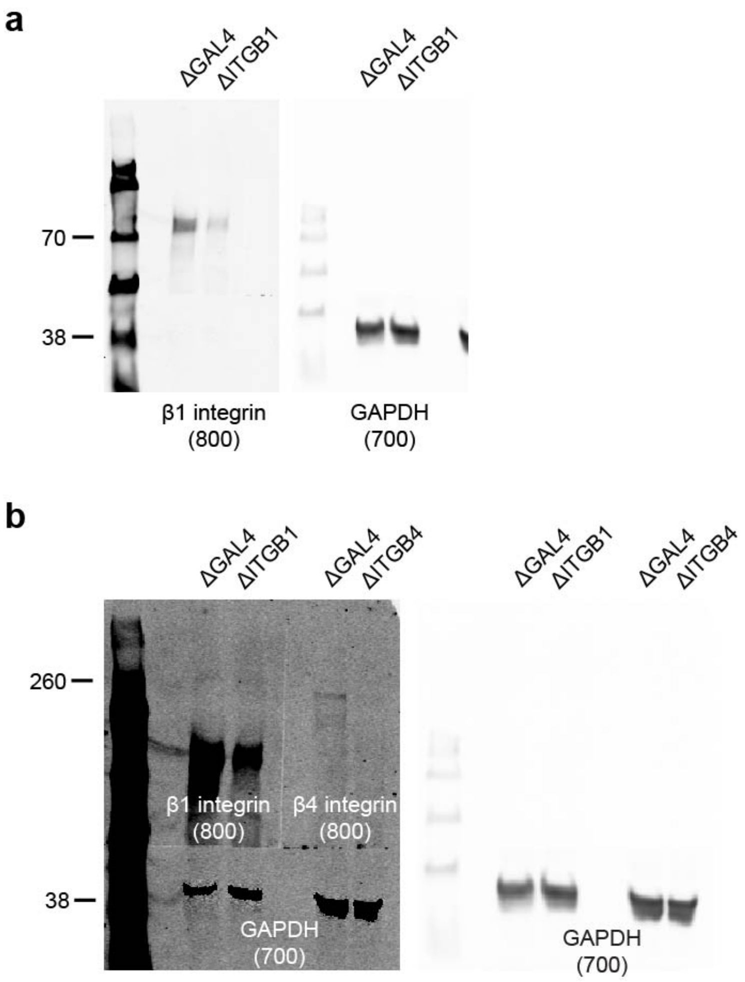

Supplementary Figure 3. Uncropped Western blots from KOs. (a) Lower exposure β_1 integrin and GAPDH blots. **(b)** Higher exposure β_4 integrin and GAPDH blots.
